# Smoking prevalence, exposure to secondhand smoke, and factors associated with smoking among medical, dental, and pharmacy students in a public university in Malaysia

**DOI:** 10.18332/tid/185751

**Published:** 2024-07-05

**Authors:** Rashidi Mohamed, Isa Naina-Mohamed, Jaya Kumar, Nadzmi Teh

**Affiliations:** 1 Department of Family Medicine, Faculty of Medicine, National University of Malaysia, Bangi, Malaysia; 2 Department of Pharmacology, Faculty of Medicine, National University of Malaysia, Bangi, Malaysia; 3 Department of Physiology, Faculty of Medicine, National University of Malaysia, Bangi, Malaysia

**Keywords:** medical, dental, pharmacy, secondhand smoking

## Abstract

**INTRODUCTION:**

Smoking among medical, dental, and pharmacy students is an issue in every university worldwide, which will impact future smoking cessation services as they are future healthcare providers. This study investigates the smoking prevalence, exposure to secondhand smoke, and factors associated with smoking among medical, dental, and pharmacy students at a public university in Malaysia.

**METHODS:**

The self-administered online survey utilized in this cross-sectional study was derived from the Global Health Professions Students Survey (GHPSS), which involved medical, dental, and pharmacy students. A total of 328 participants completed a questionnaire from June to August 2022, with a response rate of 91.1%.

**RESULTS:**

The overall smoking prevalence was 4.6% among the medical, dental, and pharmacy students who participated in this study; 46.7% of current smokers were exposed to secondhand smoke at home compared to 17.6% of non-smokers (p=0.011); and 66.7% of smokers were exposed to secondhand smoke in public compared to 40.3% of non-smokers (p=0.043). In all, 99.1% of respondents supported the smoking ban and 46.7% of current smokers supported the smoking ban in discos/bars/pubs, compared to 82.0% of non-smokers (p=0.002). Of the participants, 96.6% received lessons on the danger of tobacco, and 65.5% received smoking cessation training. Among factors associated with current smoking was gender; male students had a 19-fold higher likelihood of smoking than female students (adjusted odds ratio, AOR=19.25; 95% CI: 4.25–87.19, p<0.001). In addition, home exposure to secondhand smoke was four times more common for current smokers (OR=4.11; 95% CI: 1.43–11.79, p=0.009).

**CONCLUSIONS:**

Although smoking prevalence was low among the students in this study, there was a higher percentage of them exposed to secondhand smoke at home and in public.

## INTRODUCTION

Tobacco use remains a problem internationally despite ongoing awareness campaigns. Globally, the death rate from non-communicable diseases is five times higher than infectious diseases, and tobacco is responsible for 5 million deaths annually^1^. Approximately 21.3% of Malaysians aged >15 years were smokers; higher prevalence in Malay (7.1%) than in Chinese (4.4%) and Indians (1.5%)^2^. A higher number of smokers will cause a strain on the healthcare system as tobacco use is a risk factor for many problems, including cardiovascular diseases^3^. Tobacco use is associated with mortality; every week, tobacco kills 403 men and 119 women in Malaysia^4^. The country’s finance is heavily burdened by tobacco use as a significant part of the the national gross domestic product (GDP) is channeled to the healthcare related to smoking^5^.

Healthcare providers, including doctors, dentists, and pharmacists, are vital in tobacco control programs and serve as tobacco-free ambassadors for society^6^. Health professionals play an important role in counseling patients in quit-smoking clinics, based on the Transtheoretical Model of behavioral change^7^. However, smoking health professionals are far less likely to provide cessation services^8^. Higher education establishments in Malaysia have been gazetted as smoke-free environments, and smoking on campus has been banned since 2004.

Europe has a diverse incidence of smoking among the general population, ranging from as low as 14.4% in Iceland to 43.4% in Greece^9^. A study done among dental students in two universities in Europe found that Italian students have a higher prevalence of smoking than their Polish counterparts (42% vs 28%), higher than the general Italian population of 21%^10^.

In Egypt, 12% of medical students smoke, compared to 22% of the country’s population^11^. In this study, among the smokers, 27% were males, and 3% were females. In Nepal, the smoking prevalence of medical students was 30%, lower than the general population at 37%^12^. At the same study, 49.6% of the students were exposed to secondhand smoke at home and in public. In Laos, the prevalence is 5.1% compared to 35% of the general population; the study found male gender and exposure to secondhand smoke as factors associated with smoking^13^.

In a previous study in 2006, 2.4% of medical students at the University Putra Malaysia were smokers, and all were males^14^. A recent study done in six universities in Klang Valley yielded that 18.7% of university students in Malaysia are smokers. No previous studies have compared the smoking rates among Malaysian students studying medicine, dentistry, and pharmacy^14-17^. It was reported that in Malaysia, more than half of the secondary school adolescents were exposed to SHS, and this was higher among male and current smokers.

This study investigates the smoking prevalence, secondhand smoke exposure and factors associated with smoking among medical, dental and pharmacist students at Universiti Kebangsaan Malaysia (UKM). This information will contribute to ascertaining the magnitude of smoking and the development of smoking cessation programs for medical, pharmacist and dental students.

## METHODS

This cross-sectional study was conducted in the Faculties of Medicine, Pharmacy, and Dentistry, at a public university in Malaysia. The inclusion criteria were undergraduate students from the above three faculties, were literate in English, and who consented. Those who refused to participate were excluded from the study.

The sample size was calculated for students from each faculty, using Sample Size Calculation version 1.7.1 based on the two proportions formula^18^. Based on a previous study, the sample size for medical students, using a smoking prevalence of 6.20% with a 95% confidence interval and 0.05 significance level, was 119^14^ and taking into account a 15% dropout rate, the corrected sample size for medical students was 140. For dental students, the sample size required was 106^16^ and the corrected sample size was125. Finally, the sample size for pharmacy students was 80^13^ with corrected sample size 95^19^. The total sample size combining medical, dental, and pharmacy students was 360. A list of students was obtained from the academic office of each department. Participants from each department were selected through simple random sampling using a random generator, Kutools, in Microsoft Excel.

### Study tool

The primary tool in this study was the Global Healthcare Professions Student Survey (GHPSS), developed by the World Health Organization (WHO), the Centers for Disease Control and Prevention (CDC), and the Canadian Public Health Association (CPHA). It is part of the Global Tobacco Surveillance System (GTSS), which collects data through the three surveys: Global Youth Tobacco Survey (GYTS), the Global School Personnel Survey (GSPS), and GHPSS^20^. It was developed to collect data on tobacco use and cessation counseling among health professional students in WHO member states. The GHPSS consists of 42 core questions divided into six sections. The first section contains 3 questions on demography (age, gender, and ethnicity); the second section has 9 questions about cigarette smoking, the use of other tobacco products such as chewing tobacco, shisha, and electronic cigarettes; the third section has 4 questions on secondhand smoke inhalation at home and in public and awareness of smoking policy in school; the fourth section has 11 questions on students’ attitude towards tobacco control; the fifth section has 8 questions on evaluating any smoking cessation attempt; and the sixth section has 7 questions on the providence of education and training on tobacco cessation to patients in the school curriculum. This validated questionnaire has been used in a previous study in Malaysia^16^. The outcome variables are current smokers and non-smokers (ex-smokers and never smokers). Students who have smoked cigarettes at least once in the last 30 days are considered current smokers, students who once smoked cigarettes but no longer do so at the time of the study are considered ex-smokers, and never smokers are students who had never smoked in their lifetime^13,21^. The variables tested were age, gender, ethnicity, exposure to secondhand smoke, and received tobacco education. Exposure to secondhand smoke at home was defined as students who had household secondhand smoke exposure within seven days preceding the survey. Exposure to secondhand smoke in public was defined as students who were exposed to secondhand smoke in public within seven days preceding the survey. Received tobacco education was defined as students who answered yes to having received lessons on the danger of smoking.

### Data collection

Data collection was done during the COVID-19 pandemic. A liaison officer was appointed for each faculty to distribute the questionnaire to the participants. The liaison officers were given brief information about the questionnaire. Participants were informed of the study via WhatsApp messenger, and liaison officers distributed online questionnaires via the Jotform platform. Participants were encouraged to approach the liaison officers or the researchers if they had doubts or questions regarding the research. Participants were required to complete every question in each section to proceed to the next section. Finally, the researchers collected the completed online questionnaire via the Jotform platform.

### Data analysis

A total of 328 participants completed the questionnaires, from June to August 2022, with a response rate of 91.1%. Data analysis was performed using IBM SPSS Statistics version 27. Data are described as frequencies and percentages for the participants’ demographic characteristics, smoking behaviors, attitude towards tobacco control, belief in the roles of health professionals, smoking ban policy in university, exposure to tobacco at home and in public, and the provision of tobacco education. Bivariate analysis using the two-tailed Pearson’s chi-squared and Fisher’s exact tests was used to test the association between current smokers and non-smokers. Statistical significance was set at p<0.05. In addition, logistic regression analysis was employed to ascertain the factors associated with smoking among the students. There were no missing data as the online questionnaire required the participants to complete all questions.

## RESULTS

### Sociodemographic characteristics

A total of 360 questionnaires were distributed, with 328 participants consenting and responding to the questionnaire, a response rate of 91.1%. Of these, 130 out of 140 medical students (92.9% response rate) responded, corresponding to 39.6% of the total sample size; 112 out of 125 dental students responded (89.6% response rate) or 34.1% of the total sample size; and 86 out of 95 (90.5%) pharmacy students responded or 26.2% of the total sample size.

The sociodemographic characteristics of the participants were as follows: 236 (72.0%) participants were females, and 92 (28.0%) were males. In terms of ethnicity, 185 participants were Malays (56.4%), 58 Chinese (17.7%), 55 Indian (16.8%), and 30 other ethnicities (9.1%). In terms of age, there were 5 (1.5%) participants aged 21 years, 123 (37.8%) 22 years, 112 (34.1%) 23 years, 82 (24.5%) 24 years, and 5 (1.5%) aged 25 years.

### Prevalence of smoking

The prevalence of current smoking among the students was 4.6%. The highest prevalence was among the dental students (5.4%), followed by medical students (4.6%), and pharmacy students (3.5%). The prevalence of ex-smokers was 7.9%. The majority of the respondents (87.5%) were never smokers. Table 1 provides more details on smoking prevalence. In addition, 9.8% of participants said they had used other tobacco products, like chewing tobacco, snuff, bidis, cigars, pipes, shisha/hookah, rolled-on cigarettes, or electronic cigarettes.

**Table 1:** Smoking prevalence of medical, dental and pharmacy students

*Categories*	*Medicine (N=130) n (%)*	*Dentistry (N=112) n (%)*	*Pharmacy (N=86) n (%)*	*Total (N=328) n (%)*
**Current smokers**	6 (4.6)	6 (5.4)	3 (3.5)	15 (4.6)
**Ex-smokers**	5 (3.8)	9 (8.0)	12 (14.0)	26 (7.9)
**Never smokers**	119 (91.5)	97 (86.6)	71 (82.6)	287 (87.5)

### Exposure to secondhand smoke

Overall, 18.9% of respondents were exposed to secondhand smoke at home, and 41.5% were exposed to secondhand smoke in public. Table 2 shows that current smokers were more likely to be exposed to secondhand smoke at home than non-smokers (46.7% vs 17.6%, respectively, p=0.011). In addition, 66.7% of current smokers were exposed to secondhand smoke in public in the past seven days before the study compared to 40.3% of non-smokers (p=0.043). However, there were no statistically significant differences between faculties in exposure to secondhand smoke at home and in public.

**Table 2:** Participants exposed to secondhand smoke at home and in public

*Exposure*	*Smokers (N=15) n (%)*	*Non-smokers (N=323) n (%)*	*Total (N=328) n (%)*	*p*
**During the past 7 days, at my home, someone smoked in my presence**	7 (46.7)	55 (17.6)	62 (18.9)	0.011^b^
**During the past 7 days, in public, someone smoked in my presence**	10 (66.7)	126 (40.3)	192 (41.5)	0.043^a^

a Chi-squared test.

b Fisher’s exact test.

### Attitude towards tobacco control, belief in health professionals’ roles, and awareness of the school’s smoking ban policy

The majority of respondents responded positively to tobacco control despite their smoking status, as shown in Table 3. For example, 99.1% of participants (n=325) agreed to a smoking ban in restaurants and public places. In addition, 92.3% of participants supported the complete banning of tobacco product advertising. However, regarding the smoking ban in discos/bars/pubs, 46.7% of current smokers agreed, compared to 82.0% of non-smokers (p=0.002). Regarding the role of health professionals, 80.0% of current smokers agreed that health professionals serve as role models for patients, the public, and their patients, compared to 91.7% of non-smokers; 99.4% of participants agreed on tobacco cessation training for health professionals. Both smokers and non-smokers support that health professionals should routinely advise patients to quit smoking (93.3% vs 98.1%, respectively), and 97.8% of participants agreed that health professionals should advise patients to quit other tobacco products. In all, 99.7% of participants agreed that health professionals have a role in advising patients to quit smoking, and 93.6% of participants agreed that the chance to quit would increase with health professionals’ advice. Most respondents (80.8%) knew of their school’s smoking ban in buildings and clinics. However, fewer respondents (74.7%) reported enforcement of the smoking ban.

**Table 3:** Attitude towards tobacco control, the belief of healthcare professionals’ role and awareness of school smoking among smokers and non-smokers

	*Smokers (N=15) n (%)*	*Non-smokers (N=323) n (%)*	*Total (N=328) n (%)*	*p ^a^*
**Agreement with the following**				
Banning tobacco sales to adolescents	262 (83.7)	12 (80.0)	274 (83.5)	0.721
Complete banning of advertising of tobacco products	289 (92.3)	15 (100.0)	304 (92.7)	0.613
Smoking ban in restaurants	14 (93.3)	311 (99.4)	325 (99.1)	0.131
Smoking ban in discos/bars/pubs	7 (46.7)	262 (83.7)	269 (82.0)	0.002
Banning smoking in public places	15 (100)	310 (99.0)	325 (99.1)	1.000
Tobacco cessation training for health professionals	15 (100)	311 (99.4)	326 (99.4)	1.000
Health professionals serve as role models	12 (80.0)	287 (91.7)	299 91.2)	0.137
Health professionals routinely give quitting advice	14 (93.3)	307 (98.1)	321 (97.9)	0.282
Health professionals routinely advise patients to quit other tobacco products	15 (100)	306 (97.8)	321 (97.9)	1.000
Role of health professionals in advising patients for smoking cessation	15 (100)	312 (99.7)	327 (99.7)	1.000
Increase the chance of quitting with health professionals’ advice	15 (100)	293 (93.6)	308 (93.9)	0.611
**Awareness of the school’s smoking policy**				
Does your school have an official policy banning smoking in school? (yes)	14 (93.3)	251 (80.2)	265 (80.8)	0.439
Is your school’s official smoking ban for school buildings and clinics enforced? (yes)	13 (86.7)	232 (74.1)	245 (74.7)	0.539

a Fisher’s exact test.

### Provision of tobacco education

Most students had received lessons on the danger of smoking regardless of smoking status (93.3% of smokers and 96.8% of non-smokers), and 82.4% of non-smokers and 80.0% of smokers received lessons on the reasons of smoking (Table 4). In addition, most smokers and non-smokers were taught the importance of recording tobacco use history (100% vs 97.8%, respectively), and 64.5% of non-smokers and 86.7% of smokers were trained in smoking cessation. Most smokers (93.3%) and non-smokers (92.7%) learned the importance of providing educational quitting materials. Similarly, most students knew of nicotine replacement therapies for smoking cessation (96.6% of all participants). However, only 39.6% of participants heard of antidepressant use in smoking cessation.

**Table 4:** Participants who received tobacco education, by smoking status

*Tobacco education*	*Smokers (N=15) n (%)*	*Non-smokers (N=323) n (%)*	*Total (N=328) n (%)*	*p*
Received lessons on the dangers of smoking	14 (93.3)	303 (95.3)	317 (96.6)	0.407^b^
Received lessons on reasons of smoking	12 (80.0)	258 (82.4)	270 (82.3)	0.735^b^
Taught on the importance of recording tobacco use history	15 (100)	306 (97.8)	321 (97.9)	1.000^b^
Trained in smoking cessation	13 (86.7)	202 (64.5)	215 (65.5)	0.078^a^
Learnt the importance of providing educational quitting materials	14 (93.3)	290 (92.7)	304 (92.7)	1.000^b^
Heard of nicotine replacement therapies	15 (100)	302 (96.5)	317 (96.6)	1.000^b^
Heard of antidepressant use in smoking cessation	6 (40.0)	124 (39.6)	130 (39.6)	0.976^a^

a Chi-squared test.

b Fisher’s exact test.

### Factors associated with current smoking

Current smokers were more likely to be exposed to secondhand smoke in the home (OR=4.11; 95% CI: 1.43–11.79, p=0.009). No statistically significant association between age (OR=1.91; 95% CI: 0.66– 5.53, p=0.233), ethnicity (OR=0.88; 95% CI: 0.31–2.48, p=0.806), secondhand smoke in public (OR=2.97; 95% CI: 0.99–8.89, p=0.052) and received training on the danger of smoking (OR=2.16; 95% CI: 0.26–18.11, p=0.476) with current smoking, was found in this study. After multivariable logistic regression was tested using variables with p<0.25 from simple logistic regression, only gender remained significant, with male students more likely to be current smokers than female students (AOR=19.25; 95% CI: 4.25–87.19, p<0.001). In addition, age and exposure to secondhand smoke in public and at home were not significant (Table 5).

**Table 5:** Factors associated with current smoking

*Variables*	*Smokers (N=15) n (%)*	*Simple logistic regression*		*Multiple logistic regression*	
		*OR (95% CI)*	*p*	*AOR (95% CI)*	*p*
**Sex**					
Male	13 (14.1)	19.25 (4.25–87.19)	<0.001	19.25 (4.25–87.19)	<0.001
Female ®	2 (0.8)	1		1	
**Exposure to secondhand smoke in the home during the past 7 days**					
No ®	8 (2.4)	1		NS	
Yes	7 (2.1)	4.11 (1.43–11.79)	0.009		NS
**Exposure to secondhand smoke in public during the past 7 days**					
No ®	5 (1.5)	1			
Yes	10 (3.0)	2.97 (0.99–8.89)	0.052	NS	NS
**Age** (years)					
≤23 ®	9 (3.7)	1		NS	NS
≥24	6 (6.9)	1.91 (0.66–5.53)	0.233		
**Received training on the dangers of smoking**					
Yes ®	14 (4.3)	1	0.476		
No	1 (0.3)	2.16 (0.26–18.11)			
**Ethnicity**					
Malays	8 (2.4)	0.88 (0.31–2.48)	0.806		
Non-Malays ®	7 (2.1)	1			

AOR: adjusted odds ratio; multivariable logistic regression was tested using only variables with p<0.25 from simple logistic regression. NS: not significant. ® Reference categories.

## DISCUSSION

### Smoking prevalence

Findings from this study revealed a low (4.6%) overall prevalence of smoking among medical, dental, and pharmacy students in the university. This is lower than the smoking prevalence of 21% among the general population in Malaysia^2^. Moreover, it is lower when compared to smoking prevalence among healthcare professional students in Germany (25.6%), Egypt (12%), China (7.0%), and Laos (5.1%) ^11,13,22,23^. The rate of ex-smokers in this study was 7.9%, which was lower compared to 35.2% in Laos^13^.

The 4.6% prevalence of smoking amongst the students of this study, is lower than a study in 2006, of 6.2% amongst students at Universiti Putra Malaysia, and 5.3% in Penang Medical College^14,17^ in 2011. Even though the prevalence of smoking in our study was low, there was a need to find out the factors associated with smoking among these future physicians. Studies showed that stress was a prominent reason these students smoke^11,17^.

The prevalence of smoking among dental students in this study was 5.4%, lower than the 6% in Laos^13^, and higher than the 2.4% of dental students in a another study^16^. The latter study, conducted in six universities, recruited fewer dental students than our study, which might explain the higher percentage of smoking among the dental students in our study. There is a need to address the smoking problem, as a study in Turkey found that dental students had the misconception that their cigarette use was just a bad habit that could be stopped at anytime^24^.

The prevalence of smoking among pharmacy students in this study was 3.5%, higher than 1.2% found in a study conducted in a public and a private university in Malaysia^25^. This could be because that study had a lower response rate of 46.9% compared to our study. In addition, the previous study found that pharmacy students smoke because of stress related to their studies.

Our study had a higher prevalence of smoking among pharmacists and dental students and a lower prevalence of smoking among medical students when compared to other local studies^14,16,17,25^. One of the strategies to address the smoking issue among healthcare professional students is the provision of tobacco education and cessation interventions^26^. Most of the participants in this study had received lessons on the danger of tobacco. In addition, cigarette smoking has largely been replaced with e-cigarettes among university students^15^. In this study, more respondents (9.8%) reported using other tobacco products, including e-cigarettes, than cigarette use. Most studies found that stress was a common reason why students smoke, including those from the medical, dental, and pharmacy faculties. Therefore, there is a need to curb smoking and other tobacco product use among healthcare professional students, despite the low prevalence found in this study, to ensure the success of quit-smoking programs for the patients.

### Exposure to secondhand smoke

More than one-third of the participants (41.5%) reported having secondhand smoke exposure in public, despite the smoking ban policy in public places, including universities, which was introduced in 2004; more recently, there has been a smoking ban in eateries since 2019^27,28^. Thus, to reduce smoking prevalence among these students, enforcement of the smoking ban policy in the university must be increased, as 36.3% of participants of our study reported that the smoking ban was not enforced.

In this study, students exposed to secondhand smoke at home were likelier to smoke. This finding supports data from other schools in Italy, Europe, and Asian countries like China and Laos^10,13,22^. However, the exposure to secondhand smoke at home in this study (18.9%) was lower than that (31.0%) from the national survey^2^. An explanation for this finding was that most clinical year students stay in hostels where smoking was prohibited. Due to the limited places to smoke after the smoking ban policy in public and eateries, smokers were likelier to smoke at home, as shown by the higher prevalence of exposure to secondhand smoke at home compared to the workplace, according to the national survey^2^. Therefore, this could be the reason for the student’s exposure to secondhand smoke at home.

Secondhand smoke includes mainstream smoke, the smoke emitted from the burning end of a cigarette or other tobacco products, and sidestream smoke, the smoke that a smoker exhales^29^. Students exposed to secondhand smoke had similar health consequences to those who actively smoke. Similar changes in reduced lung capacity and increased lipid peroxidation were found among active and passive smokers^30^. Unfortunately for non-smokers, besides the mainstream smoke, the sidestream smoke they inhale contains more vessel-damaging toxins. The same meta-analysis showed that the higher the exposure to secondhand smoke, the higher the risk of developing stroke^31^. Secondhand smoke is a risk factor for various diseases, including atherosclerosis. In addition, if left untreated, the built-up plaque could cause coronary artery disease, leading to young onset acute coronary syndrome in the future^3,32^.

As many as 29% of medical students in China were depressed, and 11% had suicidal intentions^33^. Furthermore, academic pressure was found to be a factor in mental health problems among these students in the meta-analysis. In addition, about one-third of medical students at Universiti Sains Malaysia (USM) reported having stress due to academic requirements^34^. Apart from physical health consequences, secondhand smoke exposure affects mental health. This harmful smoke exposure is linked to an increased risk of developing stress, depression, and suicidal ideation^35^. An explanation is that the nicotine inhaled from secondhand smoke affects the brain’s neurotransmitters regulating mood, particularly in adolescents whose brains are still developing^36^. Furthermore, nicotine exposure during the young age of this group was related to lifelong nicotine dependence. As a result, this study’s medical, dental, and pharmacy students belong to the adolescent group at risk of mental health conditions, including schizophrenia, depression, and anxiety, linked with nicotine from secondhand smoke exposure.

Secondhand smoke affects students’ cognitive performance^37^. Health professional students face constant stress from academic pressure worsened by secondhand smoke exposure. Having their studies affected leads to an increase in stress, which is associated with a smoking habit. Therefore, it is necessary to reduce secondhand smoke exposure as it triggers a vicious cycle of stress and smoking among these students. The best method is introducing stricter smoking ban policies and higher fines in the university, as a preventive measure against health consequences because tobacco-free policy in universities was shown to reduce secondhand smoke exposure^38^.

### Attitude towards tobacco control and provision of tobacco education

The majority of participants supported tobacco control policies and health professionals’ role in smoking cessation regardless of smoking status, similar to a previous local study among dental students^16^. An explanation for this is that the respondents knew about the danger of cigarette smoke as most of them had received lessons on the topic in this study, which supports previous data from other schools^10,12,13,16,22^. However, smokers are less supportive of the smoking ban in pubs, discos, or bars, as in a previous study^16^. These places were viewed as social cues to smoke and entertainment, as they were restricted from smoking in other places in public^39^. In addition, fewer current smokers supported health professionals as role models compared to non-smokers, similar to a previous study^13^. Therefore, regardless of smoking status, there is a need to empower these students to be an example to society for a healthy lifestyle in line with the World Health Organization’s recommendation^6^.

Both smokers and non-smokers participants reported receiving training on the reasons people smoke; as future health providers, they are expected to provide cessation counseling by applying the factors of smoking in the 5R intervention^40,41^. However, 65.5% of participants reported being trained in smoking cessation, lower than in previous studies^13,16^. In the country, there is a lack of standardized guidelines for smoking cessation curricula, and respondents who just started their clinical year might not have the chance to receive the training. Efforts should be made to empower future health providers in smoking cessation as apart from dentists, doctors, and pharmacists, are team members of quit smoking services in the country^42^. Another important finding is that less than half of the participants (39.6%) knew of antidepressant use in smoking cessation. This finding supports data from previous studies among dental students in Malaysia and healthcare professional students in Laos^13,16^. However, it is lower than that (60.2%) from a study among medical students in Nepal^12^. As future health professionals, students need to be taught about the availability of pharmacotherapy for smoking cessation, which increases quitting rate success, and of antidepressants^41^.

### Factors associated with smoking

According to the survey, male students were 19 times more prone to smoke than female students. This finding was similar to other studies locally and in other Asian countries^12,13,16,17,22^. Male gender predominance might be due to culturally defined gender roles prominent in Asian culture^43^. Another factor in the higher smoking rate among males was having parents or family members who smoked^44^. Furthermore, in the same study, peer influence was a major factor in initiating smoking, while religion was a strong factor in quitting smoking, similar to another local study^17^. Thus, smoking cessation programs should consider these factors to ensure success. The low prevalence of female smokers in this study mirrored the decreasing prevalence of female smokers in the national survey^2^.

Another finding from this study is that household exposure to secondhand smoke is four times more common for current smokers. Again, this finding is similar to previous studies^10,13^. The students know the danger of tobacco smoke from the lessons received at the university. This was proven because most students agreed to a smoking ban to limit secondhand smoke exposure regardless of smoking status. There is a need to address secondhand smoke exposure at home as those exposed to secondhand smoke are more likely to develop an increased tobacco use habit^45^. Additionally, in another local study, parents or family members who smoke, influence health professional students’ smoking outcomes^17^. However, in the same study, family members were also shown as a reason to quit smoking. Campaigns focusing on the health consequences of secondhand smoke need to be enhanced so that students can enjoy a smoke-free environment at home. Smokers who smoke at home risk harming their family members and should be offered quit-smoking referrals and pharmacotherapy as a harm reduction strategy^40^. In a local study, smokers treated with nicotine replacement therapy were three times more likely to cease smoking^7^.

### Strengths and limitations

The strength of this study was it used a sample size that was adequate, with a high response rate (91.1%). The tool for this study was a validated questionnaire by WHO, CDC, and CHPA, which targeted the population selected in the study.

However, this study has its limitations. First, the types of tobacco products used by the student group, the frequency of smoking, and the presence of nicotine addiction were not fully measured. Moreover, due to its cross-sectional design, this study cannot attribute causality but can only identify associations. Second, students might not have disclosed their smoking status, despite the anonymity of the questionnaire and the confidentiality of the data. Third, this study only included participants from UKM, one of the 33 universities in Malaysia that offer medical, dental and pharmacy courses. Thus, future studies should consider enrolling participants from other universities to investigate the overall smoking prevalence in this population. Fourth, this study is cross-sectional and so describes the population at a single point in time, compared to a cohort study that studies the continuation of the condition. Finally, the COVID-19 pandemic would have influenced the results of this study, as the pandemic may have affected the smoking characteristics of the students.

## CONCLUSIONS

Despite the low prevalence of smoking among medical, dental, and pharmacy students in this study, there was a high percentage of participants exposed to secondhand smoke at home and an even higher percentage exposed to secondhand smoke in public. In addition, male gender and exposure to secondhand smoke at home were associated with smoking among these students. Therefore, there is a need to increase enforcement of the smoking ban policy, especially in the students’ accommodation at the university, to reduce further the smoking prevalence among these students.

## Data Availability

The data supporting this research are available from the authors on reasonable request.
